# Inversion of a large-scale circuit model reveals a cortical hierarchy in the dynamic resting human brain

**DOI:** 10.1126/sciadv.aat7854

**Published:** 2019-01-09

**Authors:** Peng Wang, Ru Kong, Xiaolu Kong, Raphaël Liégeois, Csaba Orban, Gustavo Deco, Martijn P. van den Heuvel, B.T. Thomas Yeo

**Affiliations:** 1 Department of Electrical and Computer Engineering, ASTAR-NUS Clinical Imaging Research Centre, Singapore Institute for Neurotechnology and Memory Networks Program, National University of Singapore, Singapore, Singapore.; 2 Center for Brain and Cognition, Department of Technology and Information, Universitat Pompeu Fabra, Barcelona, Spain.; 3 Institució Catalana de la Recerca i Estudis Avançats, Universitat Barcelona, Barcelona, Spain.; 4 Brain Center Rudolf Magnus, Department of Psychiatry, University Medical Center Utrecht, Utrecht, Netherlands.; 5 Athinoula A. Martinos Center for Biomedical Imaging, Massachusetts General Hospital, Charlestown, MA, USA.; 6 Centre for Cognitive Neuroscience, Duke-NUS Medical School, Singapore, Singapore.; 7 NUS Graduate School for Integrative Sciences and Engineering, National University of Singapore, Singapore, Singapore.

## Abstract

We considered a large-scale dynamical circuit model of human cerebral cortex with region-specific microscale properties. The model was inverted using a stochastic optimization approach, yielding markedly better fit to new, out-of-sample resting functional magnetic resonance imaging (fMRI) data. Without assuming the existence of a hierarchy, the estimated model parameters revealed a large-scale cortical gradient. At one end, sensorimotor regions had strong recurrent connections and excitatory subcortical inputs, consistent with localized processing of external stimuli. At the opposing end, default network regions had weak recurrent connections and excitatory subcortical inputs, consistent with their role in internal thought. Furthermore, recurrent connection strength and subcortical inputs provided complementary information for differentiating the levels of the hierarchy, with only the former showing strong associations with other macroscale and microscale proxies of cortical hierarchies (meta-analysis of cognitive functions, principal resting fMRI gradient, myelin, and laminar-specific neuronal density). Overall, this study provides microscale insights into a macroscale cortical hierarchy in the dynamic resting brain.

## Teaser

Converging evidence from biophysical modeling, magnetic resonance imaging, and histology reveals a large-scale cortical gradient.

## INTRODUCTION

There is converging microscale and macroscale evidence of hierarchical organization in the primate cerebral cortex, with sensory and association regions at opposite ends of the hierarchy (*1*). In terms of microscale evidence, cortical regions have been ordered into an anatomical hierarchy based on the laminar patterns of inter-regional projections (*1*, *2*). Gene expression patterns and magnetic resonance imaging (MRI) estimates of cortical myelin appeared to vary along this anatomical hierarchy (*3*). Macroscale evidence has also arisen from task-evoked functional MRI (fMRI) and lesion studies (*4*, *5*), as well as from resting-state functional connectivity (RSFC) (*6*). However, there are few studies exploring the links between microscale and macroscale cortical hierarchies.

A powerful approach to bridge microscale and macroscale brain organization is the simulation of large-scale biophysical models of coupled brain regions (*7*–*9*). This approach models the local neural dynamics in cortical regions by parsimonious but biophysically plausible neural mass or neural field models (*8*, *10*). The local models are coupled together by anatomical connections estimated from diffusion MRI or invasive tract tracing. The resulting large-scale circuit models can be used to simulate complex neural dynamics that are transformed into realistic resting-state fMRI (rs-fMRI) signals via an additional biophysical hemodynamic model (*11*, *12*). In contrast to statistical algorithms [e.g., independent component analysis (ICA)] widely used to interrogate macroscale brain organization, the dynamical properties of these large-scale circuit models are governed by parameters with physical interpretation (e.g., membrane resting potentials), some of which could be empirically measured using cellular neurophysiology or histology. Therefore, large-scale circuit models can potentially provide insights into the microscale organization of the dynamic resting brain using only macroscale MRI measurements.

However, most previous large-scale circuit studies assumed that local circuit properties are the same across brain regions (*13*–*15*). Since different brain regions have distinct microscale and macroscale properties (*16*), assuming identical parameters across brain regions is overly simplistic. Here, we relaxed the influential dynamic mean-field model [MFM; (*14*)], so that the recurrent connection strength and excitatory subcortical inputs were free to be different across cortical regions. A previously published optimization framework based on dynamic causal modeling [DCM; (*17*, *18*)] was then adapted to automatically optimize the relaxed MFM (rMFM) parameters. The resulting rMFM yielded a markedly better fit to empirical RSFC from new out-of-sample participants (53% improvement over the original MFM), suggesting the importance of allowing circuit parameters to differ across brain regions. We then investigated the relationships between the rMFM parameters with other potential proxies of cortical hierarchy using an atlas of resting-state networks (*19*), a meta-analysis of cognitive functions (*20*), the first principal RSFC gradient (*6*), T1-weighted/T2-weighted (T1w/T2w) MRI estimate of cortical myelin (*21*), and histological estimates of cell density/size (*22*).

The contributions of this study are twofold. First, we automatically inferred the parameters of a biologically plausible MFM with heterogeneous circuit properties. Although the rMFM made no assumption of the existence of a cortical hierarchy, the resulting model parameters revealed a large-scale cortical gradient. At one end of the cortical gradient, sensorimotor networks had strong recurrent connections and excitatory subcortical inputs. At the other end of the cortical gradient, the default network had weak recurrent connections and excitatory subcortical inputs. This contrasts with studies that have simply assumed local circuit parameters to follow an anatomical hierarchy (*23*, *24*) or relied on statistical approaches not explicitly linked to biophysical mechanisms (*3*, *6*).

Second, the two rMFM parameters provided complementary information for differentiating the levels of the cortical hierarchy. At one end of the hierarchy, recurrent connection strength differentiated among sensorimotor and attention networks and was strongly associated with other macroscale and microscale proxies of cortical hierarchies (cognitive functions, principal RSFC gradient, myelin, and cell density). At the other end of the cortical hierarchy, subcortical inputs differentiated among limbic, control, and default networks and was only weakly associated with the principal RSFC gradient. Overall, this study provides a computational framework for understanding the microscale foundations of macroscale cortical organization.

## RESULTS

### Automatic optimization of rMFM parameters significantly improves agreement between simulated and empirical RSFC

**Fig. 1:**
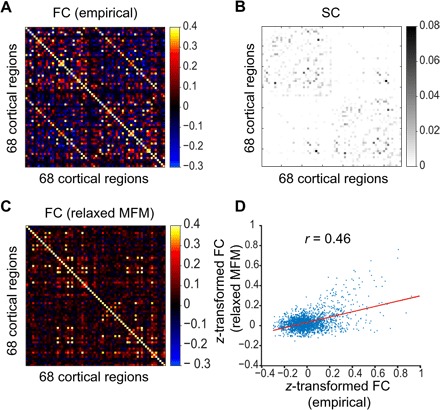
Automatic optimization of rMFM parameters yields stronger agreement between empirical and simulated RSFC. (**A**) 68 × 68 empirical FC matrix of 68 ROIs from HCP test set (*n* = 226). (**B**) 68 × 68 SC matrix from the HCP test set. (**C**) Simulated 68 × 68 FC matrix using SC matrix from the test set and rMFM parameters estimated from the HCP training set (*n* = 226). (**D**) Correlation between inter-region simulated FC and inter-region empirical FC (ignoring diagonal elements of the matrices). Correlation between SC and empirical FC in the test set was 0.30. Correlation between simulated and empirical FC was 0.46.

Four hundred fifty-two participants from the Human Connectome Project (HCP) S500 release (*25*) were randomly divided into training (*n* = 226) and test (*n* = 226) sets. Following previous work (*14*), the Desikan-Killiany cortical parcellation (*26*) with 68 regions of interest (ROIs) was used to compute group-averaged RSFC and structural connectivity (SC) matrices from the training and test sets separately. Figure 1 (A and B) shows the 68 × 68 group-averaged FC and SC matrices, respectively, from the test set.

Neural dynamics of the 68 ROIs were simulated with the MFM (*14*). Here, we highlight the intuitions behind the MFM; details of the model are found in Materials and Methods. The MFM assumes that the neural dynamics of each ROI is driven by four components: (i) recurrent (intraregional) input, (ii) inter-regional inputs, (iii) excitatory subcortical input, and (iv) neuronal noise. There are “free” parameters associated with each component. First, *w* is the strength of recurrent connections within a region; a higher *w* increases the amount of recurrent input current relative to other inputs. Second, the inter-regional inputs depend on the neural activities of the other cortical ROIs and the connection strength between ROIs. The connectional strength is determined by the corresponding SC entries scaled by a global constant *G*. Thus, a higher *G* increases the relative strength of inter-regional inputs. Third, *I* is the excitatory subcortical input (in nanoampere). Fourth, the neuronal noise is assumed to be Gaussian with noise amplitude σ.

While the original MFM assumed all four parameters to be constant across brain regions, here, the model was relaxed to allow the recurrent connection *w* and subcortical input *I* to be different across ROIs, resulting in 138 parameters. We will refer to this extended model as rMFM. Given each ROI’s neural activity, the hemodynamic model (*11*, *12*) was used to simulate blood oxygen level–dependent (BOLD) fMRI at each ROI. The simulated fMRI could then be used to compute a 68 × 68 FC matrix. Following previous work (*14*), agreement between the simulated and empirical FC matrices was defined as the Pearson’s correlation between the two matrices (ignoring the diagonal and nonunique entries) and was used as an index of model fit.

A previous optimization framework for inverting neural mass models for magnetoencephalography (*18*) was adapted to automatically estimate the rMFM parameters by maximizing the agreement between simulated and empirical FC (see Materials and Methods for details). By applying the optimization algorithm to the training set, rMFM parameters (*w* and *I* in each ROI, as well as *G* and σ) were estimated. The estimated model parameters were used together with the test set SC to generate simulated FC (Fig. 1C). Across 1000 simulations, correlation between simulated and empirical FC in the test set (Fig. 1D) was, on average, 0.46, with an SD of 0.016.

The same procedure was repeated for the MFM (i.e., *w* and *I* were constrained to be the same across ROIs). Across 1000 simulations, correlation between simulated and empirical FC in the test set was, on average, 0.30, with an SD of 0.016. This is not better than the baseline correlation of 0.30 between SC and empirical FC in the test set.

### Sensory-motor systems exhibit strong recurrent connections and excitatory subcortical input, while the default network exhibits weak recurrent connections and excitatory subcortical input

**Fig. 2:**
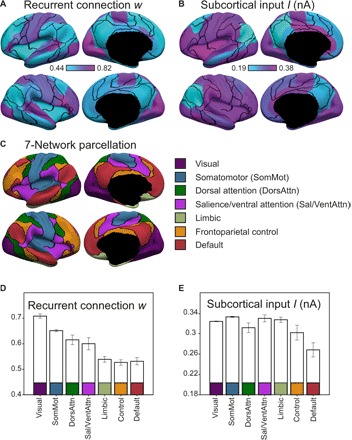
Strength of recurrent connections *w* and subcortical inputs *I* in 68 anatomically defined ROIs and their relationships with seven resting-state networks. (**A**) Strength of recurrent connection *w* in 68 anatomically defined ROIs. (**B**) Strength of excitatory subcortical input *I* in 68 anatomically defined ROIs. Parcels correspond to the 68 Desikan-Killiany ROIs (*26*). Black boundaries correspond to the boundaries of seven canonical resting-state networks (*19*). (**C**) Seven resting-state networks (*19*). (**D**) Strength of recurrent connections *w* in the seven resting-state networks. (**E**) Strength of subcortical input *I* in the seven resting-state networks. Regions within sensory-motor systems exhibited strong recurrent connections and excitatory subcortical input, while those within the default network exhibited weak recurrent connections and excitatory subcortical input.

The above results (Fig. 1) underline the importance of accounting for regional heterogeneity of local circuit properties in biophysical modeling. The question is whether the heterogeneity pattern might be biologically plausible or meaningful. To address this question, Fig. 2 (A and B) shows the spatial distribution of the estimated recurrent connection strength *w* and excitatory subcortical input *I* for the 68 anatomically defined Desikan-Killiany parcels. The black lines correspond to the boundaries of seven canonical resting-state networks (*19*). For reference, Fig. 2C shows the seven resting-state networks with the same black boundaries as in Fig. 2 (A and B).

While the resting-state network boundaries (black boundaries in Fig. 2) do not exactly align with the anatomically defined parcels [see further discussion in (*19*)], there was a notable correspondence between the resting-state networks and the estimated rMFM parameters. In particular, the sensorimotor regions appeared to exhibit strong recurrent connections (Fig. 2A), while regions of the default network appeared to exhibit weak subcortical inputs (Fig. 2B).

To quantify this phenomenon, we transferred the estimated rMFM parameters from the 68 Desikan-Killiany parcels to the seven resting-state networks. Briefly, we performed the “transfer” as follows. First, we extracted 51 ROIs from the spatially distributed resting-state networks. For example, the default network (red in Fig. 2C) was extracted into 10 ROIs across different lobules. Second, we transferred the rMFM parameter estimates from the 68 Desikan-Killiany parcels to all vertices (from the underlying cortical meshes) comprising each anatomical parcel. Third, we then averaged the parameter estimates across all vertices constituting each of the 51 resting-state ROIs.

Figure 2D shows the strength of recurrent connection *w* across the resting-state networks. Visual and somatomotor systems exhibited the strongest recurrent connections, while limbic, control, and default networks exhibited the weakest recurrent connections. Dorsal attention and salience/ventral attention networks exhibited intermediate recurrent connections.

Figure 2E shows the strength of the excitatory subcortical input *I* across the resting-state networks. The default network exhibited the weakest subcortical input, while the visual, somatomotor, dorsal attention, salience/ventral attention, and limbic networks exhibited the strongest subcortical inputs. The control network exhibited intermediate subcortical inputs.

### Regions with high recurrent connection strength are involved in sensory-motor processing, while those with low recurrent connection strength are involved in higher cognitive functions

The correspondences between the estimated rMFM parameters and the resting networks (Fig. 2), as well as the well-known correspondence between resting and task networks, suggest that the regional variation in recurrent connection strength and subcortical inputs might reflect cortical processing hierarchies, whereby information might flow from sensory regions to association regions. To make this connection more explicit, we considered 12 cognitive components from a previous large-scale meta-analysis of 10,449 task-evoked functional experiments (*20*). The cognitive components were associated with distinct patterns of brain activity and task profiles. On the basis of the top 5 tasks associated with each component [table S1; (*20*)], these components could be interpreted as being involved in various neural processes, ranging from sensory-motor functions (e.g., “hand,” “auditory,” and “visual”) to higher cognitive functions (e.g., “working memory” and “internal mentation”).

**Fig. 3:**
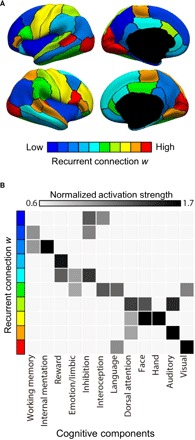
Relationship between recurrent connection strength *w* and BrainMap cognitive components. (**A**) 68 Desikan-Killiany ROIs are grouped into 10 zones spanning low to high recurrent connection strength *w*. (**B**) Twelve cognitive components derived from meta-analysis of 10,449 experiments (*20*) are ordered on the basis of the average normalized activation strength within each of the 10 zones. Zones with high recurrent connection strength were involved in sensory perception and motor actions (visual, auditory, hand, and face), while those with low recurrent connection strength were involved in cognitive functions, such as working memory, internal mentation, and reward.

To explore the relationships between the estimated rMFM parameters and the cognitive components, we grouped the 68 Desikan-Killiany ROIs into 10 zones, ranging from low to high recurrent connection strength *w* (Fig. 3A). The normalized activation strength associated with each cognitive component was averaged within each of the 10 zones (see details in Materials and Methods). Figure 3B shows the average activity of the 10 zones. The cognitive components were ordered on the basis of the average normalized activation strength within each of the 10 zones.

Zones with high recurrent connection strength exhibited greater brain activity associated with sensory perception and motor actions (e.g., “face,” hand, visual, auditory, etc.). On the other hand, zones with low recurrent connection strength were involved in cognitive functions, including working memory, internal mentation, “reward,” “emotion/limbic,” and “inhibition” (Fig. 3B), which were consistent with known cognitive functions of limbic, control, and default networks (Fig. 2D).

The relationship between subcortical input and cognitive functions (fig. S1) was also consistent with the resting-state network analysis (Fig. 2E). More specifically, zones with high subcortical inputs appeared to exhibit a range of cognitive functions (e.g., emotion/limbic, face, hand, visual, “dorsal attention,” etc.) that were consistent with the fact that sensorimotor, attentional, and limbic networks exhibited uniformly high subcortical inputs. On the other hand, zones with low subcortical inputs were involved in working memory and internal mentation, which are cognitive functions typically associated with the control and default networks.

These results (Figs. 2 and 3) suggest that regional variation in rMFM parameters (especially recurrent connection strength) might be more closely related to traditional processing hierarchies. We will now consider two other proxies of cortical processing hierarchies: the first principal RSFC gradient (*6*) and cortical myelin content (*3*).

### Strength of recurrent connections and subcortical inputs is associated with the first principal connectivity gradient

**Fig. 4:**
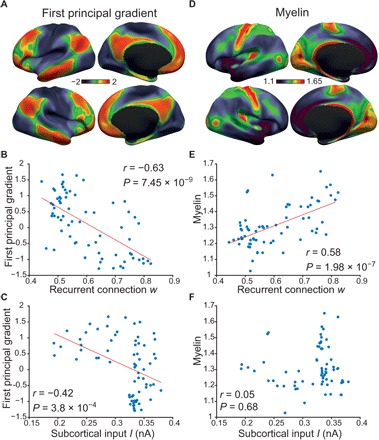
Associations of estimated rMFM parameters (strength of recurrent connection *w* and subcortical input *I*) with the first principal RSFC gradient and relative myelin content. (**A**) First principal RSFC gradient obtained by diffusion embedding of the human connectome (*6*). (**B**) Association between recurrent connection *w* and first principal gradient. (**C**) Association between subcortical input *I* and first principal gradient. (**D**) T1w/T2w ratio map of estimated myelin content (*21*). (**E**) Association between recurrent connection *w* and myelin. (**F**) Association between subcortical input *I* and myelin.

One proxy of cortical processing hierarchy is the first principal RSFC gradient, obtained by applying a nonlinear dimensionality reduction algorithm to estimate principal components (“gradients”) that accounted for the greatest RSFC variance within the cerebral cortex (*6*). The first principal gradient was interpreted to represent a processing hierarchy anchored by sensorimotor regions at one end and the default network at the other end [Fig. 4A; (*6*)].

To investigate the relationship between the first principal RSFC gradient and the estimated rMFM parameters, the first principal gradient values were averaged within each Desikan-Killiany ROI and correlated against the estimated recurrent connection strength *w* (Fig. 4B) and subcortical input *I* (Fig. 4C). The principal gradient was strongly correlated with the recurrent connection strength (*r* = −0.63, *P* = 7.45 × 10^−9^) and weakly correlated with the subcortical input (*r* = −0.42, *P* = 3.8 × 10^−4^).

We note that the sign of the correlation is arbitrary because the direction of the first principal gradient is arbitrary. Here, we followed the convention of the original study: low principal gradient values in sensorimotor regions and high principal gradient values in the default network. However, it would be equivalent to reverse the direction of the principal gradient so that sensorimotor regions exhibited high principal gradient values, while the default network exhibited low principal gradient values.

### Recurrent connection strength is positively associated with estimates of cortical myelin

The T1w/T2w ratio has been widely used as an estimate of relative cortical myelin content [Fig. 4A; (*21*)]. Burt and colleagues (*3*) suggest that the T1w/T2w myelin estimate is a good macroscale proxy of microscale cortical processing hierarchy. To investigate the relationship between cortical myelin and the estimated rMFM parameters, we averaged the relative myelin content within each Desikan-Killiany ROI and correlated against the estimated recurrent connection strength *w* (Fig. 4B) and subcortical input *I* (Fig. 4C). Recurrent connection strength *w* was positively correlated with relative myelin content (*r* = 0.58, *P* = 1.98 × 10^−7^), while there was no association between subcortical input *I* and relative myelin content.

### Strength of recurrent connections is positively associated with increased neuronal density

Neuronal density (neuron count per cubic millimeter) and neuronal size of the Desikan-Killiany parcels were previously estimated by van den Heuvel and colleagues (*22*) based on the cytoarchitectonic work of von Economo and Koskinas (*27*). To investigate the relationship between neuronal density (or size) and estimated rMFM parameters, we first note that von Economo and Koskinas did not explicitly differentiate between left and right hemispheres in their mapping. Visual inspection of Fig. 2 suggests that estimates of the recurrent connection strength *w* and excitatory subcortical input *I* appeared relatively symmetric across the hemispheres. Therefore, the rMFM parameter estimates were averaged between corresponding left and right anatomical parcels and then correlated with estimates of neuronal density and neuronal size.

**Fig. 5:**
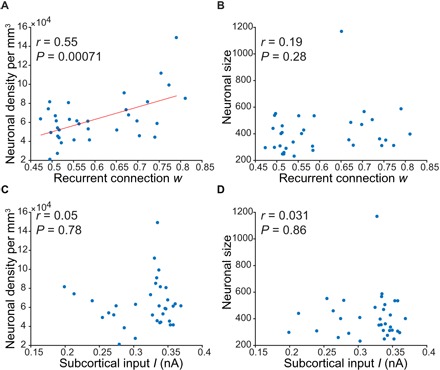
Associations between estimated rMFM parameters (strength of recurrent connection *w* and subcortical input *I*) and cytoarchitectonic measures (neuronal density and neuronal size) averaged across all cortical layers. (**A**) Association between recurrent connection w and neuronal density averaged across all cortical layers. (**B**) Association between recurrent connection w and neuronal size averaged across all cortical layers. (**C**) Association between subcortical input I and neuronal density averaged across all cortical layers. (**D**) Association between subcortical input I and neuronal size averaged across all cortical layers.

Figure 5 shows that the recurrent connection strength *w* was correlated with neuronal density averaged across all cortical layers (*r* = 0.55, *P* = 0.00071), but not with neuronal size. The excitatory subcortical input *I* was not correlated with neuronal density or neuronal size (Fig. 5). Table 1 reports all examined correlations. All bolded correlations survived a false discovery rate of *q* < 0.05. The recurrent connection strength *w* was also significantly correlated with neuronal density in cortical layers 2, 3, and 6.

**Table 1:** Pearson’s correlation between estimated rMFM parameters (recurrent connection *w* and subcortical input *I*) and cytoarchitectonic data (neuronal cell density and cell size). *P* values that survived a false discovery rate of *q* < 0.05 are bolded.

	***w***	***P***	***I***	***P***
Layer 1 density	−0.13	0.48	0.34	0.053
Layer 2 density	**0.50**	**0.0038**	0.23	0.21
Layer 3 density	**0.52**	**0.0015**	0.078	0.66
Layer 4 density	0.39	0.036	−0.10	0.60
Layer 5 density	−0.11	0.54	0.24	0.17
Layer 6 density	**0.56**	**0.00054**	0.23	0.20
Cell density averaged across all layers	**0.55**	**0.00071**	0.050	0.78
Layer 1 size	−0.29	0.090	0.026	0.88
Layer 2 size	−0.26	0.15	−0.17	0.37
Layer 3 size	0.20	0.25	−0.042	0.81
Layer 4 size	0.31	0.10	−0.052	0.79
Layer 5 size	0.26	0.14	0.13	0.46
Layer 6 size	−0.18	0.30	−0.18	0.31
Cell size averaged across all layers	0.19	0.28	0.031	0.86

### Replication with a higher-resolution parcellation and other control analyses

The above analyses were replicated using a higher-resolution Lausanne2008 parcellation with 114 cortical ROIs (*28*). More specifically, across 1000 simulations, correlation between simulated and empirical FC in the test set was, on average, 0.46, which was significantly better than the baseline correlation of 0.34 between SC and empirical FC in the test set. Similar to before, we found that sensory-motor networks exhibit relatively strong recurrent connections and excitatory subcortical input, while the default network exhibits relatively weak recurrent connections and excitatory subcortical input (fig. S2).

Just like in the case of the Desikan-Killiany analyses, Lausanne 2008 parcels with low recurrent connection strength were associated with higher cognitive functions, while parcels with high recurrent connection strength were associated with sensory perception and motor actions (fig. S3). The relationship between subcortical input and cognitive functions was less clear (fig. S4). The recurrent connection strength was also positively correlated with myelin, while both recurrent connection strength and subcortical input were negatively correlated with the first principal gradient (fig. S5).

Recurrent connection strength *w* was correlated with neuronal density averaged across all cortical layers (*r* = 0.49, *P* = 0.00012), but not with neuronal size averaged across all cortical layers (table S2). The excitatory subcortical input *I* was not correlated with neuronal density or neuronal size. The recurrent connection strength *w* was also significantly correlated with neuronal density in cortical layers 2, 4, and 6, as well as neuronal size in cortical layer 4 (table S2).

Last, we considered an alternative division of the 68 Desikan-Killiany ROIs into five cortical types (*27*). These cortical types were originally defined on the basis of laminar cytoarchitectonic patterns and have been functionally interpreted along an action-perception axis, with type 1 representing action and type 5 representing perception. Cortical types 1 to 3 appeared to exhibit low recurrent connection strength *w* and subcortical input *I* (fig. S6).

## DISCUSSION

There is a rich literature on hierarchical organization within the primate cerebral cortex (*1*, *29*, *30*). While traditional accounts of cortical hierarchies have suggested that sensory streams terminate in prefrontal areas (*5*, *31*), others have suggested that the apex of processing hierarchy is occupied by parallel, distributed association networks spanning frontal, parietal, temporal, and cingulate cortex (*32*). Under this latter account, cortical areas within each association network might occupy the same hierarchical level.

Recent human brain network literature has largely supported this network perspective. However, it also proposes that certain association networks might “control” other networks, suggesting (explicitly or implicitly) hierarchical differentiation among association networks (*29*, *30*). For example, Cole and colleagues (*30*) suggested that the frontoparietal (control) network shifts its connectivity patterns for adaptive execution of different tasks. Menon and Uddin (*29*) suggested that the salience network is involved in detecting behaviorally relevant stimuli and switching between large-scale networks to facilitate access to attention and working memory resources and initiate motor systems. Margulies and colleagues (*6*) have proposed that the default network is at one end of the cortical processing hierarchy, allowing it to process abstract information not related to direct sensory stimuli.

Our results provide evidence for a large-scale hierarchy in the human cerebral cortex, with the default network and sensorimotor systems occupying opposing ends. Although the hierarchy is consistent with a recent study (*6*), our large-scale computational modeling framework enabled further elaborations and insights at the microscale level. More specifically, regional variation in recurrent connection strength was associated with all hierarchical proxies, including cognitive functions, first RSFC principal gradient, myelin, and cell density. On the other hand, regional variation in subcortical inputs was only weakly associated with the first principal RSFC gradient.

The recurrent connection strength and subcortical inputs were effective in differentiating opposite ends of the cortical hierarchy. More specifically, recurrent connection strength strongly differentiated among visual, somatomotor, dorsal attention, and salience/ventral attention networks (Fig. 2D), while limbic, control, and default networks were indistinguishable. Previously published hierarchical proxies (e.g., myelin and first principal RSFC gradient) exhibited the same issue [see Figure 3B of Margulies *et al*. (*6*)], which is not unexpected, given their strong associations with recurrent connection strength. On the other hand, the subcortical inputs strongly differentiated among limbic, control, and default networks (Fig. 2E). Thus, the two rMFM parameters (recurrent connectional strength and subcortical inputs) were complementary in differentiating the levels of the cortical hierarchy.

### Large-scale gradients of recurrent connection strength and subcortical inputs

At one end of the hierarchy, regions within sensorimotor resting-state networks exhibited strong recurrent connections (Fig. 2). This relationship with brain function was made more explicit by our meta-analysis, showing that regions involved in sensorimotor functions exhibited strong recurrent connections (Fig. 3). Furthermore, regions with strong recurrent connection strength also exhibited low RSFC principal gradient values and greater myelin content (Fig. 4). The strong positive association between the strength of recurrent connections and neuronal cell density (Fig. 5) suggests an underlying cellular basis for this phenomenon. Previous studies have demonstrated that brain regions with densely packed neurons are associated with specialized local processing (*33*, *34*). Consistent with this interpretation, regions within sensory-motor resting-state networks also appeared to have strong excitatory subcortical inputs (Fig. 2), which might correspond to the flow of sensory information from the external environment via subcortical relays.

At the other end of the cortical hierarchy, regions within the default network had weak excitatory subcortical inputs (Fig. 2), suggesting the lack of a direct flow of information from the external milieu. This supports the view that the default network is instead more associated with internal processing, as suggested by multiple studies on its involvement in self-generated thought, such as autobiographical memory, mind wandering, and thinking about the future (*35*). We also found the default network to have weak recurrent connections (Fig. 2). Furthermore, regions involved in internal mentation exhibited weak recurrent connections (Fig. 3). If strong recurrent connections are important for specialized local processing, then weak recurrent connections might be consistent with the default network’s putative role as a hub of transmodal information integration (*6*).

The attentional, limbic, and control networks occupy intermediate zones in the cortical hierarchy but are further differentiated when both recurrent connections and subcortical inputs were considered. For example, the dorsal attention and salience/ventral attention networks exhibited an intermediate recurrent connection strength, in contrast to the limbic and control networks, which exhibited weak recurrent connection strength similar to the default network (Fig. 2). On the other hand, the control network exhibited an intermediate level of subcortical inputs, in contrast to the limbic and attentional networks, which exhibited a strong level of subcortical inputs similar to the early sensorimotor systems. Thus, we might interpret the two attentional networks as sensorimotor processing systems situated between the early sensory (and/or late motor) systems and upstream limbic, control, and default networks. This is consistent with the role of the salience/ventral attention network in bottom-up processing of sensory information (*36*). In the case of the dorsal attention network, the regions comprising the network include medial temporal complex (MT+), lateral intraparietal (LIP) region, and the frontal eye fields (FEFs). These regions have also been considered as part of a canonical sensorimotor pathway extending from MT+ to LIP and FEF (*1*, *19*).

### Model realism

Statistical approaches (e.g., *k*-means, ICA, temporal ICA, sliding window correlations, and hidden Markov models) have been widely used to study brain network organization and dynamics (*37*). While these models have provided significant insights into the human brain, they are not meant to “mimic” actual brain mechanisms. On the other hand, biophysical models (e.g., spiking neural networks and neural mass models) are mechanistically plausible by incorporating model parameters linked to microscale characteristics (e.g., membrane resting potentials), some of which could be empirically measured using cellular neurophysiology or histology (*8*). Thus, biophysically plausible models can potentially be very useful for linking microscale circuit-level mechanisms with macroscale approaches (e.g., fMRI) widely used to study the human brain.

To illustrate the difference between statistical and biophysical models, we note that, although our hierarchy is consistent with the first RSFC principal gradient (*6*), the direction of the principal gradient is arbitrary. In Fig. 3A, we followed the convention of the original study: low principal gradient values in sensorimotor regions and high values in the default network. However, it would be equivalent to have high principal gradient values in sensorimotor regions and low values in the default network. By contrast, we cannot simply invert the recurrent connection strength values and conclude that sensorimotor regions have low recurrent connection strength and association regions have high recurrent connection strength. Thus, the rMFM has conceptual advantages over statistical models.

It is worthwhile mentioning that biophysical models lie on a spectrum of model realism. On this spectrum, the MFM is obviously less realistic than detailed spiking models but arguably more realistic than fMRI-DCM (*17*, *38*) in that the MFM is obtained by a mathematical (mean-field) reduction of a detailed microscopic model of neuronal activity, based on integration of higher-order neurons and realistic synaptic dynamics (keeping in mind that fMRI-DCM has a very different goal of estimating effective connectivity). This mean-field reduction is mathematically equivalent to the statistical mechanics approach, in which atoms are modeled as populations of atoms, rather than as individual atoms. Similarly, the complex behavior of individual neurons can be mathematically reduced to the behaviors of populations of neurons. Obviously, some information has been lost in this reduction, but importantly, a more realistic model of individual neurons is not better for answering all neuroscientific questions. For example, one could faithfully simulate a single cortical column with thousands of parameters (*39*), but such a model cannot be easily related to macroscale observations in fMRI (with current approaches).

In this study, the application of the DCM algorithm to the MFM is an advance along the biological realism spectrum. Nevertheless, it is clear that the specific MFM (*14*) used in this paper is still a significant simplification of actual biological processes. For example, the current MFM does not account for recurrent excitatory and inhibitory connections within the same region (*10*, *23*). As such, the recurrent connection strength in the rMFM can only be thought of as the superposition of both local excitatory and inhibitory effects. A clear future extension is to consider an rMFM that incorporated both recurrent excitatory and inhibitory connections.

### Model fit

Although the agreement between empirical and simulated FC markedly improved from the SF-FC baseline correlation of 0.30 to 0.46, it suggests possible room for improvement from better modeling (previous section), anatomical connectivity estimates, and/or optimization procedures. Given the highly nonlinear nature of the rMFM, we cannot rule out the possibility of other local optima that might yield a better fit between empirical and simulated FC.

It is difficult to directly compare our results with the quality of fit in the literature on simulating FC from SC. Unfortunately, many papers do not report baseline SC-FC correlations, so it is difficult to evaluate how much “work” the modeling performs beyond the SC-FC correlation baseline. For example, Honey and colleagues (*13*) achieved an agreement of 0.70 between empirical and simulated FC. However, they also reported a baseline SC-FC correlation of 0.66. Thus, our modeling results were arguably better, although the correlation between empirical and simulated FC was lower in our study.

Furthermore, most papers reported (in-sample) correlations using the same data used to develop their models. Since these papers used significantly less parameters, overfitting might be less of an issue than in our study. Nevertheless, the true (out-of-sample) correlations between empirical FC and simulated FC are almost certainly going to be less than the reported correlations.

## MATERIALS AND METHODS

### Data

We considered 452 participants from the HCP S500 release (*25*). All imaging data were collected on a custom-made Siemens 3T Skyra scanner using a multiband sequence. Each participant went through two fMRI sessions on two consecutive days. Two rs-fMRI runs were collected in each session. Each fMRI run was acquired in 2-mm isotropic resolution with a repetition time (TR) of 0.72 s and lasted for 14 min and 33 s. The structural data consisted of one 0.7-mm isotropic scan for each subject. The diffusion imaging consisted of six runs, each lasting approximately 9 min and 50 s. Diffusion weighting consisted of three shells of *b* = 1000, 2000, and 3000 s/mm^2^, with an approximately equal number of weighting directions on each shell. Details of the data collection can be found elsewhere (*25*). The HCP participants were randomly divided into training (*n* = 226) and test (*n* = 226) sets.

### Preprocessing

Details of the HCP anatomical, functional and diffusion preprocessing can be found elsewhere (HCP S500 manual). We used the rs-fMRI data that the HCP has already projected to the fsLR surface space, smoothed by 2 mm, and denoised with ICA-FIX. Following previous work on large-scale whole-brain computational modeling (*7*, *13*), the mean cortical signal was further regressed from the fMRI data. For each rs-fMRI run of each participant, the Pearson’s correlation between the mean fMRI time series of each parcel and every other parcel was computed, resulting in a 68 × 68 FC matrix. The FC matrices were averaged across runs and then averaged across subjects within the training (or test) set.

In the case of the diffusion data, white matter pathways were reconstructed using generalized Q-sampling imaging, allowing for the reconstruction of complex diffusion fiber configurations (i.e., crossing/kissing fibers) and streamline tractography. The result was a weighted 68 × 68 SC matrix for each subject, where the weight corresponded to the number of tractography streamlines between two regions. Details of the tractography procedure can be found elsewhere (*40*). To create the group-averaged SC matrix, for each entry of the matrix, if there were less than 50% of the training (or test) subjects with fibers in the entry, it was set to zero. Otherwise, the number of streamlines was averaged across training (or test) subjects with nonzero streamlines. This averaging procedure followed that of previous work (*40*). Because diffusion tractography was noisy in individual subjects, the thresholding procedure helped to remove false positives. Furthermore, the training (or test) SC matrix was scaled such that its maximal entry was equal to 0.2.

### Dynamic MFM

The MFM was derived by mean-field reduction of a detailed spiking neuronal network model (*14*) within each brain region to the following set of nonlinear stochastic differential equations

$$\begin{array}{c} {\dot{S}}_{i}=-\frac{S_{i}}{\tau_{s}}+r(1-S_{i})H(x_{i})+\sigma v_{i}(t)\\ H(x_{i})=\frac{ax_{i}-b}{1-\text{exp}(-d(ax_{i}-b))}\\ x_{i}=wJS_{i}+GJ\underset{j}{\Sigma }C_{ij}S_{j}+I \end{array}$$

where *x*_*i*_, *H*(*x*_*i*_), and *S*_*i*_ denote the total input current, the population firing rate, and the average synaptic gating variable at the *i*-th cortical region, respectively. The total input current *x*_*i*_ is determined by the recurrent connection strength *w*, the excitatory subcortical input *I*, and inter-region information flow. *C*_*ij*_ corresponds to the SC between the *i*-th and *j*-th cortical regions, thus controlling the strength of information flow between the two cortical regions. The global constant *G* scales the strength of information flow from other cortical regions to the *i*-th cortical region, relative to the recurrent connection and subcortical inputs. Following previous work (*14*), the value of synaptic coupling *J* was set to be 0.2609 (nA). Parameter values for the input-output function *H*(*x*_*i*_) were set to be *a* = 270 (n/C), *b* = 108 (Hz), and *d* = 0.154 (s). The kinetic parameters for synaptic activity were set to be *r*= 0.641 and τ_*s*_ = 0.1 (s). *v*_*i*_(*t*) is uncorrelated standard Gaussian noise, and the noise amplitude is controlled by σ.

The MFM captures the average neural dynamic behaviors of cortical regions at the level of neuronal populations using interpretable dynamic variables and physiological parameters, such as population firing rate and average synaptic gating variable. In comparison to more detailed spiking neuronal networks coupled by AMPA, NMDA (*N*-methyl-d-aspartate), and GABA (γ-aminobutyric acid) synapses, the MFM allows a comprehensive study of the relationship between the model parameters and large-scale brain dynamics with a relatively low parametric complexity (*7*, *14*).

The simulated neural activities *S*_*i*_ are fed to the Balloon-Windkessel hemodynamic model (*17*) to simulate the BOLD signals for each ROI. Briefly, synaptic activity *S*_*i*_ in each ROI causes an increase in vasodilatory signal *z*_*i*_. Inflow *f*_*i*_ responds in proportion to this signal with concomitant changes in blood volume *v*_*i*_ and deoxyhemoglobin content *q*_*i*_. The equations relating these biological processes are as follows

$$\begin{array}{c} {\dot{z}}_{i}=S_{i}-\kappa z_{i}-\gamma (f_{i}-1)\\ {\dot{f}}_{i}=z_{i}\\ \tau {\dot{v}}_{i}=f_{i}-{v_{i}}^{1/\alpha }\\ \tau {\dot{q}}_{i}=\frac{f_{i}}{\rho }[1-{\left(1-\rho \right)}^{1/f_{i}}]-q_{i}{v_{i}}^{1/\alpha -1} \end{array}$$

where ρ = 0.34 is the resting oxygen extraction fraction. The kinetic parameters rate of signal decay κ = 0.65 (s^−1^), rate of elimination γ = 0.41 (s^−1^), hemodynamic transit time τ = 0.98 (s), and Grubb’s exponent α = 0.32 followed previous work (*14*). Given *q*_*i*_ and *v*_*i*_, the BOLD signal is given by (*11*, *12*)

$${\text{BOLD}}_{i}=V_{0}[k_{1}(1-q_{i})+k_{2}(1-\frac{q_{i}}{v_{i}})+k_{3}(1-v_{i})]$$

where *V*_0_ = 0.02 is the resting blood volume fraction, and (*k*_1_, *k*_2_, *k*_3_)are a set of parameters dependent on magnetic field strength and a number of acquisition-dependent parameters. The equations of *k*_*i*_ are as follows (*12*)

$$\begin{array}{c} k_{1}=4.3\vartheta_{0}\rho \text{TE}\\ k_{2}=\varepsilon r_{0}\rho \text{TE}\\ k_{3}=1-\varepsilon \end{array}$$

where ϑ_0_ = 28.265*B*_0_ is the frequency offset at the outer surface of magnetized vessels and depends on the main magnetic field strength *B*_0_, which is 3*T* in the HCP dataset. At a magnetic field strength of 3*T*, the intravascular relaxation rate is *r*_0_ = 110 Hz, and the ratio between intravascular and extravascular MR signal is e = 0.47 (*12*). The echo time TE = 33.1 ms in the HCP dataset.

The MFM and hemodynamic model were simulated using Euler’s integration with a time step of 10 ms. The starting values of *S*_*i*_ in the MFM were randomly initialized. Simulation length for the BOLD signals was 16.4 min. The first 2 min of the BOLD signals were discarded, and the time series were downsampled to 0.72 s to have the same temporal resolution as the empirical BOLD signals in the HCP.

### rMFM and automatic estimation of model parameters

In the MFM, the recurrent connection strength *w* and excitatory subcortical input *I* were assumed to be the same across regions. Here, we relaxed the model, so that each region *i* had its own recurrent connection strength *w*_*i*_ and excitatory subcortical input *I*_*i*_. We refer to the resulting model as rMFM.

We optimized 138 rMFM parameters corresponding to *w*_*i*_ (for each ROI), *I*_*i*_ (for each ROI), global scaling factor *G*, and noise coefficient s by maximizing the similarity between simulated and empirical FC in the training set. The optimization was based on a previously developed algorithm for inverting neural mass models for MEG (*18*), which was, in turn, based on the expectation-maximization algorithm used in DCM (*17*). The algorithm is summarized as follows

Step 1: Initialize model parameters θ_0_ = [*w*_*i*_, *I*_*i*_, *G*, σ]^*T*^ = [0.5, 0.3, 1, 0.001].

Step 2: To ensure that the model parameters θ remain positive, the parameters were reparameterized as $\varphi =\;\text{ln}(\frac{\theta }{\theta_{0}})$. Each parameter φ was assumed to be generated from a prior distribution *N*(0, 0.25), which effectively translated into a prior mean of [0.5, 0.3, 1, 0.001] for [*w*_*i*_, *I*_*i*_, *G*, σ]^*T*^. We note that this prior distribution was sufficiently weak to cover the range of parameters (*w*, *I*, *G*, σ) used in the literature.

Step 3: Simulate BOLD signals with model parameter θ and compute the FC matrix. The upper triangular entries of the 68 × 68 simulated FC matrix were vectorized into a 2278 × 1 vector *y*_sim_ = *h*(). Let *y*_emp_ be the corresponding vector from the empirical FC matrix.

Step 4: Compute *R* = *y*_emp_ − *y*_sim_

Step 5: Numerically approximate Jacobian $J=\frac{\partial h(\varphi )}{\partial \partial }$ using Newton’s difference quotient.

Step 6: Initialize error covariance matrix *C*_*e*_ = exp(λ)*Q*, where *Q* is the 2278 × 2278 identity matrix and λ = −3.

Step 7: Update λ in the error covariance matrix *C*_*e*_ using *R* and *J* computed from previous steps [c.f. M-step in (*17*)]

$$\begin{array}{c} P=C_{e}^{-1}-C_{e}^{-1}J{\left(J^{T}C_{e}^{-1}J\right)}^{-1}J^{T}C_{e}^{-1}\\ g=-0.5\text{trace}\{PQ\}+0.5R^{T}PQPR\\ H=-0.5\text{trace}\{PQPQ\}\\ \lambda^{i+1}=\lambda^{i}+\frac{g}{H} \end{array}$$

The λ^*i*+1^ was used to update the error covariance matrix *C*_*e*_ = exp(λ^*i*+1^)*Q*, and the updated error covariance matrix was again used in the above equations to update λ until convergence.

Step 8: Update rMFM parameters φ [c.f. E-step in (*17*)]

$$\varphi^{i+1}=\varphi^{i}+{\left(J^{T}C_{e}^{-1}J+C_{\theta }^{-1}\right)}^{-1}(J^{T}C_{e}^{-1}R+C_{\theta }^{-1}\varphi^{i})$$

where *C*_θ_ = 0.25*I*_68×68_, where *I*_68×68_ is a 68 × 68 identity matrix.

Step 9: Repeat steps 3 to 8 for 512 iterations. The set of model parameters θ = θ_0_logφ resulting in the best Pearson’s correlation between the simulated and empirical FC in the training set was selected.

Step 10: Repeat steps 2 to 9 using different random initializations of φ. Each random initialization required about 36 hours using 30 central processing unit (CPU) cores for the Desikan-Killiany parcellation. Here, we considered 10 random initializations (in addition to the initialization with the prior mean in step 1). The set of model parameters with the best Pearson’s correlation between the simulated and empirical FC in the training set was selected.

### Resting-state networks

Seven resting-state networks from Yeo and colleagues (*19*) were considered. Each network was spatially distributed, e.g., the red default network (Fig. 2C) has separate components in the lateral parietal cortex, posterior cingulate/precuneus, lateral temporal cortex, medial prefrontal cortex, and so on. A connected component analysis was used to extract all the connected “islands” of each network. Small components (less than eight vertices) were reassigned to the most correlated networks. Furthermore, vertices whose neighbors belonged to a different component were removed. This resulted in a final set of 51 parcels.

It is worth noting that the use of the Desikan-Killiany atlas for the MFM followed previous applications of the MFM (*7*, *14*). The resting-state ROIs were not directly used for the MFM because some of the ROIs were small and narrow. As such, the tractography estimates would not be reliable for these small ROIs. Furthermore, results were replicated using the Lausanne 2008 parcellation, suggesting that the results were robust to the exact choice of parcellation.

### BrainMap cognitive components

Cognitive components were estimated from a large-scale meta-analysis of the BrainMap database (*20*). Each component was associated with a probability of being recruited by a task (of 83 possible task categories). The top 5 tasks for each component are found in table S1. Each component was also associated with the probability of activating a voxel. These components were mapped to fsaverage surface space (*20*). Because the probabilities must sum to one over all brain voxels, certain cognitive components (e.g., reward) that activated both cortical and subcortical structures would exhibit lower probabilities on the cortical surface. Therefore, for each component, the activation probabilities were normalized to sum to an arbitrary value of 100,000 over the entire cerebral cortex, which we will refer to as normalized activation strength.

To ensure that the numbers of cognitive components and anatomical ROIs were comparable, the 68 Desikan-Killiany ROIs were grouped into 10 zones with increasing recurrent connection strength (such that each zone has roughly the same surface area). For each cognitive component, the average normalized activation strength was computed for each of the 10 zones by summing the normalized activation strength within a zone divided by the surface area of the zone. The average normalized activation strength is plotted in Fig. 5B. The 10 zones were ordered from low (blue) to high (red) recurrent connection strength, while the 12 components were ordered on the basis of the largest normalized activation strength. For example, the visual component has the highest normalized activation strength in the red anatomical zone (highest recurrent connection strength) and was therefore assigned to the rightmost column in Fig. 5B.

### Myelin and principal gradients

The relative cortical myelin analyses used the average T1w/T2w ratio map released by the HCP (*21*). The first principal gradient map was provided by D. Margulies (*6*). In both cases, no further preprocessing was performed other than averaging the myelin and principal gradient values within each anatomical ROI.

### Code and data availability

This study followed the institutional review board guidelines of corresponding institutions. The code used in this paper is publicly available at https://github.com/ThomasYeoLab/CBIG/tree/master/stable_projects/fMRI_dynamics/Wang2018_MFMem. The HCP diffusion MRI, rs-fMRI, and myelin data are publicly available (www.humanconnectome.org/). The 51 resting-state ROIs (derived from the Yeo2011 resting-state networks) can be found here at https://github.com/ThomasYeoLab/CBIG/tree/master/stable_projects/brain_parcellation/Yeo2011_fcMRI_clustering. The von Economo data can be obtained by directly contacting M. van den Heuvel. The BrainMap cognitive components can be downloaded at https://surfer.nmr.mgh.harvard.edu/fswiki/BrainmapOntology_Yeo2015.

## Supplementary Material

http://advances.sciencemag.org/cgi/content/full/5/1/eaat7854/DC1

## SUPPLEMENTARY MATERIALS

Supplementary material for this article is available at http://advances.sciencemag.org/cgi/content/full/5/1/eaat7854/DC1 Fig. S1. Relationship between subcortical input *I* and BrainMap cognitive components. Fig. S2. Strength of recurrent connections *w* and subcortical input *I* in 114 anatomically defined ROIs and their relationships with seven resting-state networks. Fig. S3. Relationship between recurrent connection strength *w* and BrainMap cognitive components. Fig. S4. Relationship between subcortical input *I* and BrainMap cognitive components. Fig. S5. Associations of estimated rMFM parameters (using the Lausanne 2008 parcellation) with relative myelin content and first principal gradient of the human connectome. Fig. S6. Relationships between cortical types and estimated rMFM parameters. Table S1. Top 5 tasks recruiting 12 cognitive components (*20*). Table S2. Pearson’s correlation between estimated rMFM parameters (recurrent connection *w* and subcortical input *I*) using the Lausanne 2008 parcellation and cytoarchitectonic data (neuronal cell density and cell size).
