# Supplementary material for: Inversion of a large-scale circuit model reveals a cortical hierarchy in the dynamic resting human brain
Source: Sci Adv. 2019 Jan 9;5(1):eaat7854. doi: 10.1126/sciadv.aat7854 (PMC6326747; doi:10.1126/sciadv.aat7854)
Supplement: http://advances.sciencemag.org/cgi/content/full/5/1/eaat7854/DC1 [file supp_5_1_eaat7854__index.html]

Science Advances | Science Advances

## Supplementary Materials

**This PDF file includes:**

- Fig. S1. Relationship between subcortical input *I* and BrainMap cognitive components.
- Fig. S2. Strength of recurrent connections *w* and subcortical input *I* in 114 anatomically defined ROIs and their relationships with seven resting-state networks.
- Fig. S3. Relationship between recurrent connection strength *w* and BrainMap cognitive components.
- Fig. S4. Relationship between subcortical input *I* and BrainMap cognitive components.
- Fig. S5. Associations of estimated rMFM parameters (using the Lausanne 2008 parcellation) with relative myelin content and first principal gradient of the human connectome.
- Fig. S6. Relationships between cortical types and estimated rMFM parameters.
- Table S1. Top 5 tasks recruiting 12 cognitive components (*20*).
- Table S2. Pearson’s correlation between estimated rMFM parameters (recurrent connection *w* and subcortical input *I*) using the Lausanne 2008 parcellation and cytoarchitectonic data (neuronal cell density and cell size).

Download PDF

**Files in this Data Supplement:**

- Adobe PDF - aat7854\_SM.pdf
