# Supplementary material for: Inversion of a large-scale circuit model reveals a cortical hierarchy in the dynamic resting human brain
Source: Sci Adv. 2019 Jan 9;5(1):eaat7854. doi: 10.1126/sciadv.aat7854 (PMC6326747; doi:10.1126/sciadv.aat7854)
Supplement: http://advances.sciencemag.org/cgi/content/full/5/1/eaat7854/DC1 [file aat7854_SM.pdf]

Science Advances
AAAS

advances.sciencemag.org/cgi/content/full/5/1/eaat7854/DC1

Supplementary Materials for

Inversion of a large-scale circuit model reveals a cortical hierarchy in the dynamic resting human brain

Peng Wang, Ru Kong, Xiaolu Kong, Raphaël Liégeois, Csaba Orban, Gustavo Deco, Martijn P. van den Heuvel, B.T. Thomas Yeo*

*Corresponding author. Email: thomas.yeo@nus.edu.sg

Published 9 January 2019, *Sci. Adv.* **5**, eaat7854 (2019)
DOI: 10.1126/sciadv.aat7854

This PDF file includes:

Fig. S1. Relationship between subcortical input *I* and BrainMap cognitive components.
Fig. S2. Strength of recurrent connections *w* and subcortical input *I* in 114 anatomically defined ROIs and their relationships with seven resting-state networks.
Fig. S3. Relationship between recurrent connection strength *w* and BrainMap cognitive components.
Fig. S4. Relationship between subcortical input *I* and BrainMap cognitive components.
Fig. S5. Associations of estimated rMFM parameters (using the Lausanne2008 parcellation) with relative myelin content and first principal gradient of the human connectome.
Fig. S6. Relationships between cortical types and estimated rMFM parameters.
Table S1. Top 5 tasks recruiting 12 cognitive components (*20*).
Table S2. Pearson's correlation between estimated rMFM parameters (recurrent connection *w* and subcortical input *I*) using the Lausanne2008 parcellation and cytoarchitectonic data (neuronal cell density and cell size).

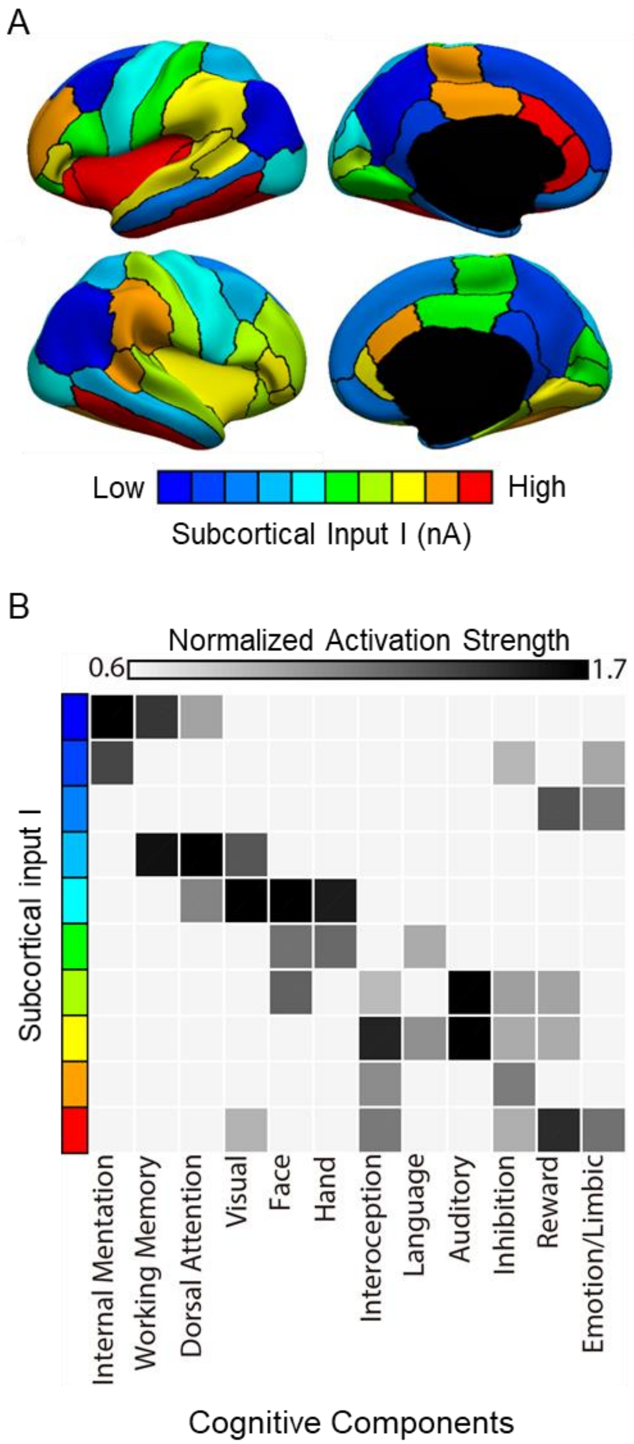

Fig. S1. Relationship between subcortical input *I* and BrainMap cognitive components. (**A**) 68 Desikan-Killiany ROIs are grouped into 10 regions spanning low to high recurrent subcortical input I. (**B**) 12 cognitive components derived from meta-analysis of 10,449 experiments (Yeo et al., 2015) are ordered based on the average normalized activation strength within each of the 10 regions. The relationship between subcortical input and cognitive functions was less clear. Regions with low subcortical input were involved in higher cognitive functions ("internal mentation" and "working memory"), while regions with high subcortical input were involved in a range of cognitive functions (e.g., "emotion/limbic", "face", "hand", "visual", "dorsal attention", etc).

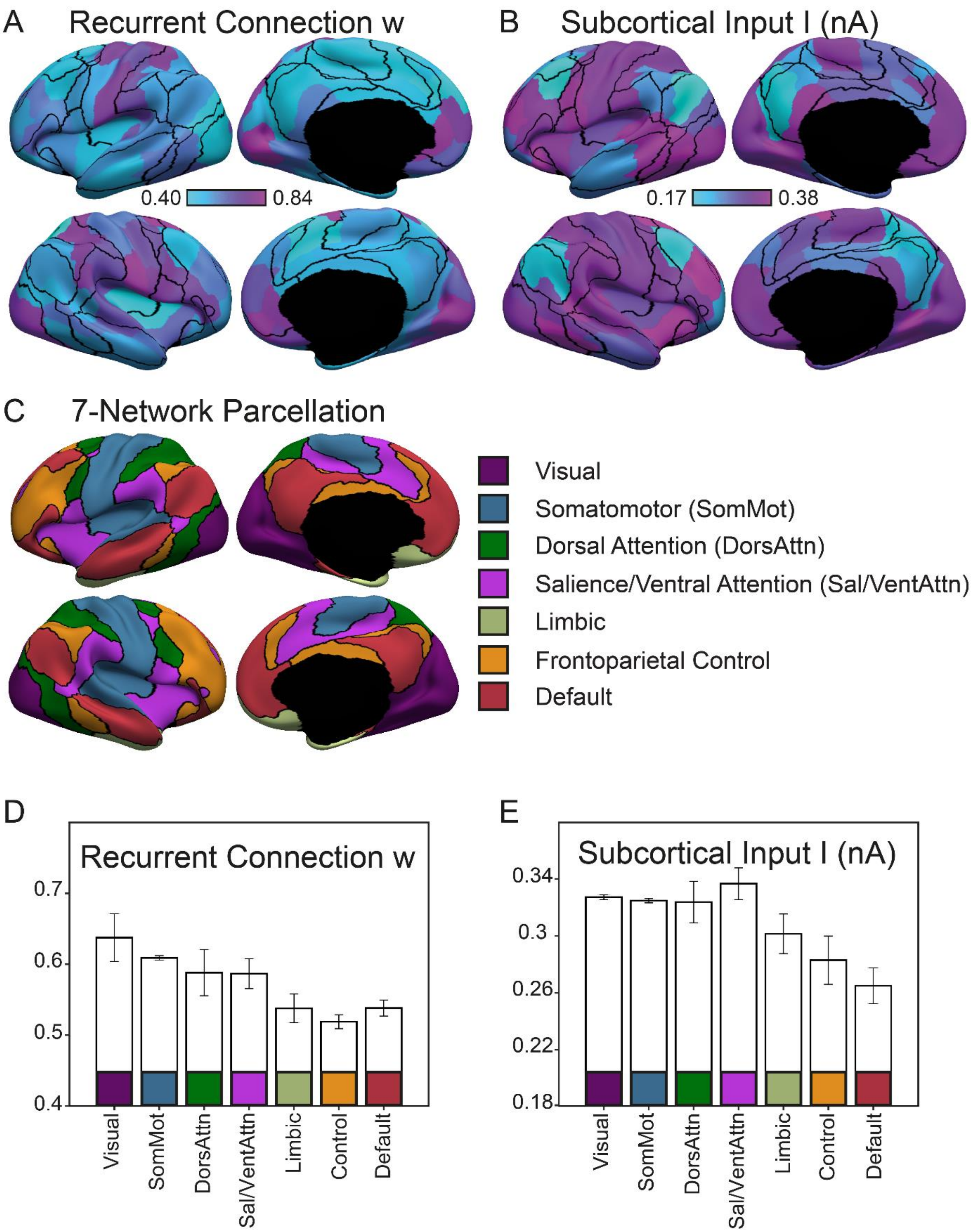

Fig. S2. Strength of recurrent connections *w* and subcortical input *I* in 114 anatomically defined ROIs and their relationships with seven resting-state networks. (**A**) Strength of recurrent connection w in 114 anatomically defined ROIs. (**B**) Strength of excitatory subcortical input I in 114 anatomically defined ROIs. Parcels correspond to the Lausanne2008 parcellation (Hagmann et al., 2008). Black boundaries correspond to the boundaries of seven canonical resting-state networks (Yeo et al., 2011). (**C**) Seven resting-state networks (Yeo et al., 2011). (**D**) Strength of recurrent connections w in the seven resting-state networks. (**E**) Strength of subcortical input I in the seven resting-state networks. Sensory-motor systems exhibited strong recurrent connections and excitatory subcortical input, while the default network exhibited weak recurrent connections and excitatory subcortical input.

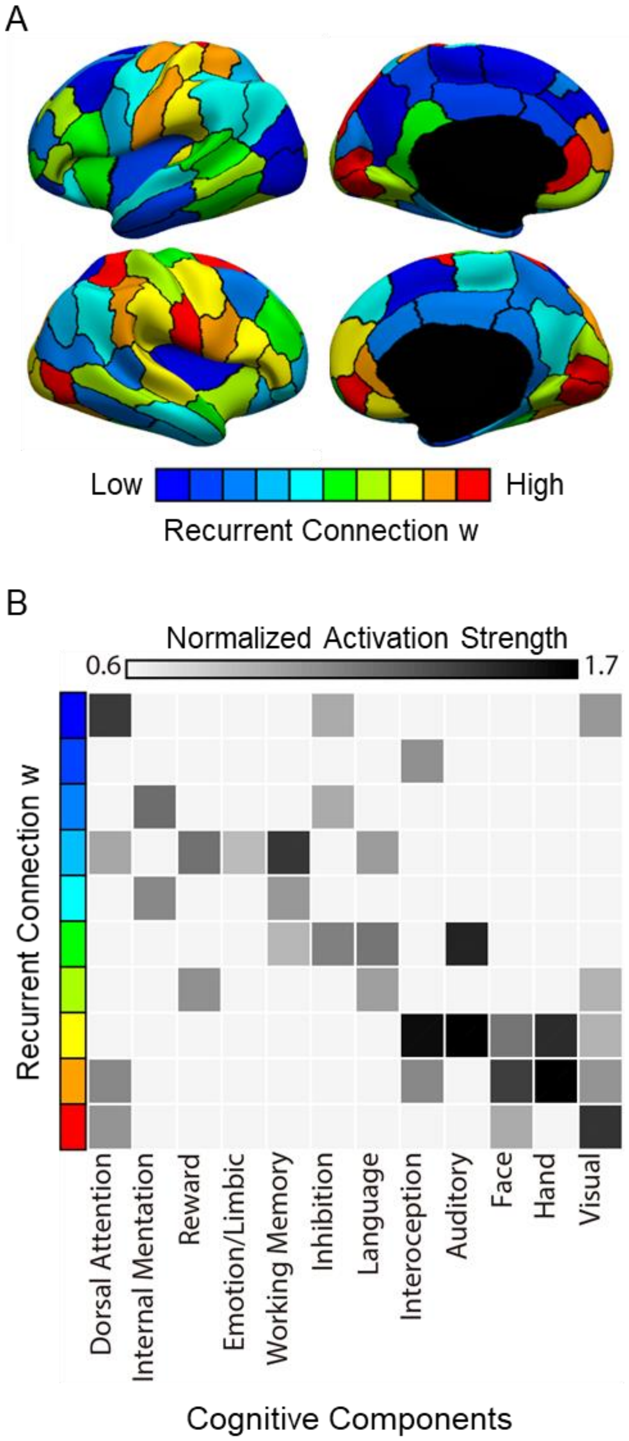

Fig. S3. Relationship between recurrent connection strength *w* and BrainMap cognitive components. (**A**) 114 Lausanne2008 ROIs are grouped into 10 regions spanning low to high recurrent connection strength w. (**B**) 12 cognitive components derived from meta-analysis of 10,449 experiments (Yeo et al., 2015) are ordered based on the average normalized activation strength within each of the 10 regions.

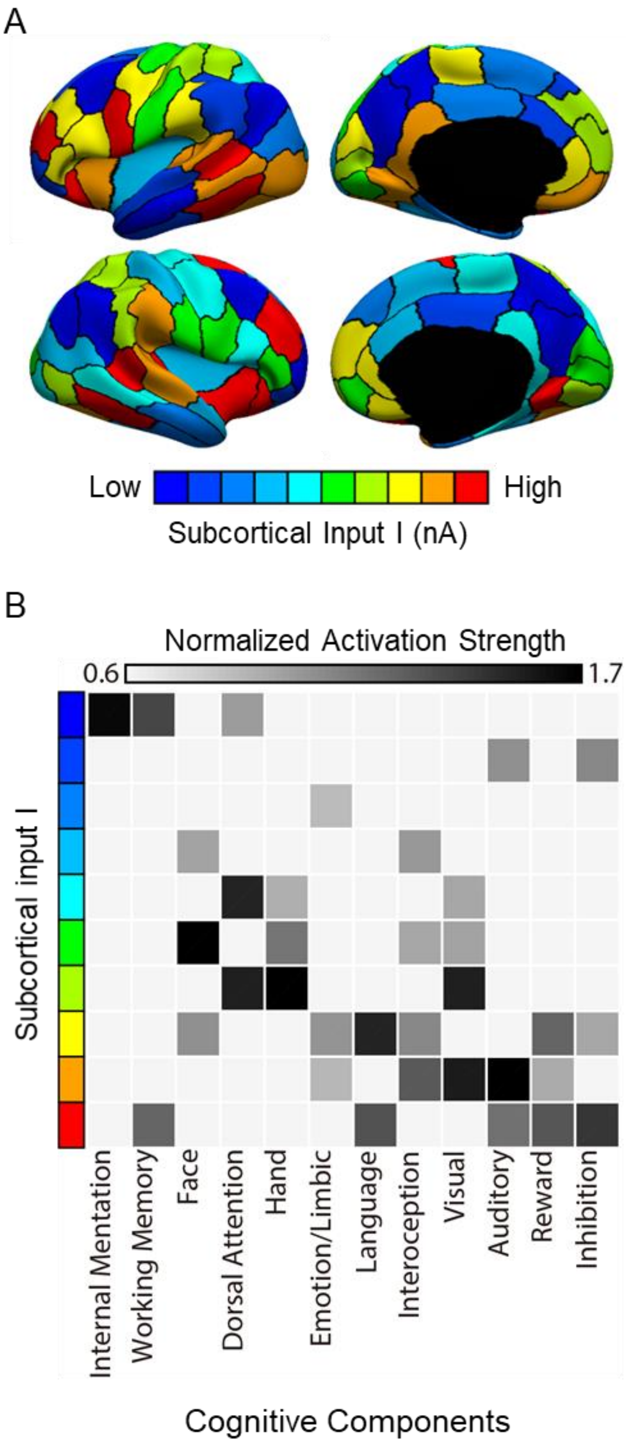

Fig. S4. Relationship between subcortical input *I* and BrainMap cognitive components. (**A**) 114 Lausanne2008 ROIs are grouped into 10 regions spanning low to high subcortical input I. (**B**) 12 cognitive components derived from meta-analysis of 10,449 experiments (Yeo et al., 2015) are ordered based on the average normalized activation strength within each of the 10 regions.

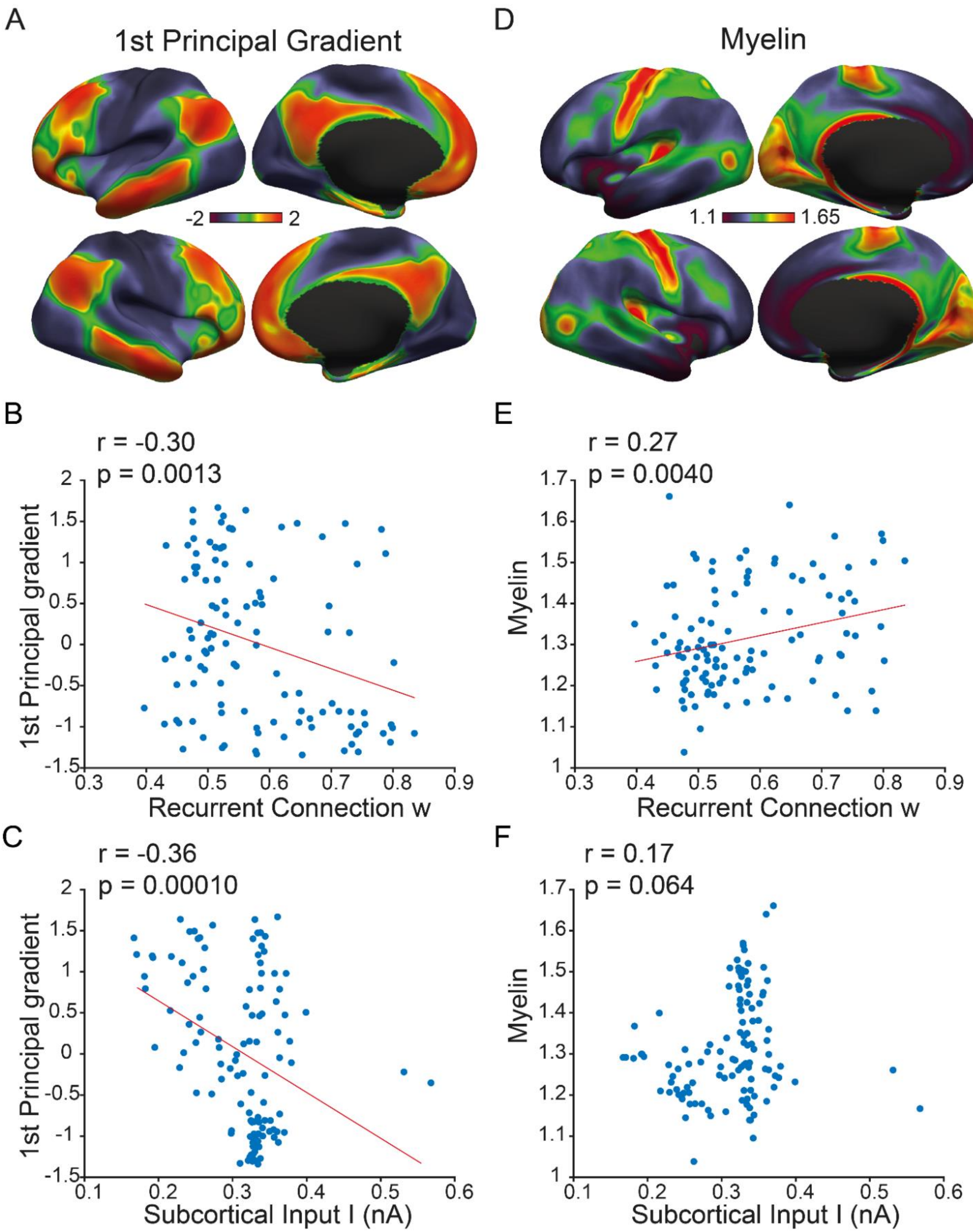

Fig. S5. Associations of estimated rMFM parameters (using the Lausanne2008 parcellation) with relative myelin content and first principal gradient of the human connectome. (**A**) Myelin map (Glasser et al., 2011) (**B**) Association between recurrent connection w and myelin (**C**) Association between subcortical input I and myelin (**D**) First principal gradient of the human connectome obtained by diffusion embedding (Margulies et al., 2016) (**E**) Association between recurrent connection w and first principal gradient (F) Association between subcortical input I and first principal gradient.

Table S1. Top 5 tasks recruiting 12 cognitive components (*20*). Top five tasks recruiting 12 cognitive components (Yeo et al., 2015). “(C)” and “(O)” indicate “covert” and “overt” respectively. “Mon”, “Discrmin” and “Attn” are short for “monitoring”, “discrimination” and “attention” respectively. “Subj Emo Pict Discrimin” is short for “Subjective Emotional Picture Discrimination”.

Hand	Face	Auditory
Vibrotactile Mon/Discrim	Recitation/Repetition (O)	Pitch Mon/Discrim
Finger Tapping	Chewing/Swallowing	Passive Listening
Grasping	Reading (Overt)	Music Comp/Production
Flexion/Extension	Flexion/Extension	Tone Mon/Discrim
TMS	Music Comp/Production	Phonological Discrim
**Visual**	**Language**	**Dorsal Attention**
Visual Pursuit/Tracking	Naming (C)	Saccades
Action Observation	Word Generation (C)	Anti-Saccades
Naming (C)	Semantic Mon/Discrimin	Pointing
Naming (O)	Reading (C)	Mental Rotation
Mental Rotation	Word Generation (O)	Visual Distractor/Attn
**Interoception**	**Inhibition**	**Working Memory**
Micturition	Flanker	WCST
Pain Mon/Discrim	Deception	Counting/Calculation
Acupuncture	Go/No-Go	n-back
Tactile Mon/Discrim	Stroop	Sternberg
Eating/Drinking	Simon	Task Switching
**Internal Mentation**	**Emotion/Limbic**	**Reward**
Theory of Mind	Face Mon/Discrimin	Reward Task
Rest	Subj Emo Pict Discrimin	Olfactory Mon/Discrimin
Fixation	Olfactory Mon/Discrimin	Eating/Drinking
Naming (O)	Passive Viewing	Classical Conditioning
Acupuncture	Classical Conditioning	Paired Associate Recall

Table S2. Pearson's correlation between estimated rMFM parameters (recurrent connection *w* and subcortical input *I*) using the Lausanne2008 parcellation and cytoarchitectonic data (neuronal cell density and cell size). P values that survived a false discovery rate of q < 0.05 are bolded.

Neuronal	w	p-value	I	p-value
Layer 1 density	0.069	0.61	0.0022	0.99
Layer 2 density	**0.47**	**0.00059**	0.17	0.23
Layer 3 density	0.25	0.057	0.20	0.14
Layer 4 density	**0.56**	**0.000030**	0.18	0.24
Layer 5 density	0.050	0.71	0.11	0.39
Layer 6 density	**0.44**	**0.00067**	0.25	0.060
Density of total layers	**0.49**	**0.00012**	0.24	0.073
Layer 1 size	-0.081	0.55	-0.0058	0.97
Layer 2 size	-0.21	0.14	-0.12	0.40
Layer 3 size	-0.16	0.24	-0.080	0.55
Layer 4 size	**0.43**	**0.0023**	0.061	0.68
Layer 5 size	0.071	0.60	-0.039	0.77
Layer 6 size	-0.20	0.14	-0.086	0.52
Size of total layers	0.0078	0.95	-0.056	0.68

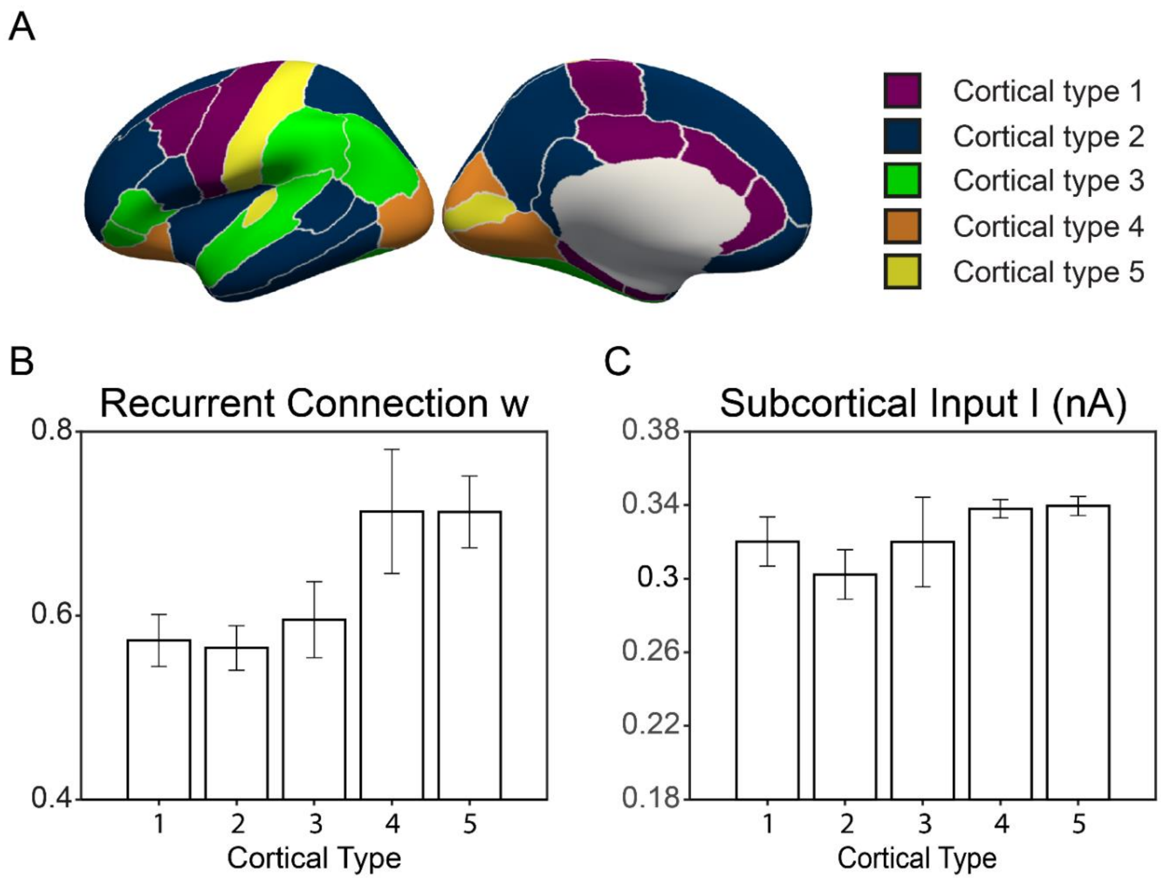

Fig. S6. Relationships between cortical types and estimated rMFM parameters. (**A**) Rough division of the 68 Desikan-Killiany ROIs into five cortical types based on Von Economo and Koskinas (1925). (**B**) Cortical types 1 to 3 exhibited low recurrent connection strength w. (**C**) Cortical types 1 to 3 exhibited low subcortical input I.
